# The neighbourhood environment and use of neighbourhood resources in older adults with and without lower limb osteoarthritis: results from the Hertfordshire Cohort Study

**DOI:** 10.1007/s10067-016-3388-5

**Published:** 2016-08-27

**Authors:** Erik J. Timmermans, Suzan van der Pas, Cyrus Cooper, Laura A. Schaap, Mark H. Edwards, Dorly J. H. Deeg, Catharine R. Gale, Elaine M. Dennison

**Affiliations:** 1 Department of Epidemiology and Biostatistics, EMGO Institute for Health and Care Research, VU University Medical Centre, De Boelelaan 1089A, 1081 HV Amsterdam, The Netherlands; 2 MRC Lifecourse Epidemiology Unit, University of Southampton, Southampton General Hospital, Southampton, UK; 3 NIHR Musculoskeletal Biomedical Research Unit, Nuffield Department of Orthopaedics, Rheumatology and Musculoskeletal Sciences, University of Oxford, Oxford, UK; 4 Department of Health Sciences, Faculty of Earth and Life Sciences, VU University Amsterdam, Amsterdam, the Netherlands; 5 Centre for Cognitive Ageing & Cognitive Epidemiology, Department of Psychology, University of Edinburgh, Edinburgh, UK; 6 School of Biological Sciences, Victoria University of Wellington, Wellington, New Zealand

**Keywords:** Neighbourhood environment, Older population, Osteoarthritis

## Abstract

This study aimed to examine the associations of perceptions of neighbourhood cohesion and neighbourhood problems and objectively measured neighbourhood deprivation with the use of neighbourhood resources by older adults with and without lower limb osteoarthritis (LLOA), and to assess whether these relationships are stronger in older persons with LLOA than in those without the condition. Data from the Hertfordshire Cohort Study were used. American College of Rheumatology classification criteria were used to diagnose clinical LLOA (knee and/or hip osteoarthritis). Use of neighbourhood resources was assessed using the Home and Community Environment instrument. Participants were asked about their perceptions of neighbourhood cohesion and neighbourhood problems. Objective neighbourhood deprivation was assessed using the Index of Multiple Deprivation score based on 2010 census data. Of the 401 participants (71–80 years), 74 (18.5 %) had LLOA. The neighbourhood measures were not significantly associated with use of resources in the full sample. A trend for a negative association between use of public transport and perceived neighbourhood problems was observed in participants with LLOA (OR = 0.77, 99 % CI = 0.53–1.12), whereas a trend for a positive association between perceived neighbourhood problems and use of public transport was found in participants without LLOA (OR = 1.18, 99 % CI = 1.00–1.39). The perception of more neighbourhood problems seems only to hinder older adults with LLOA to make use of public transport. Older adults with LLOA may be less able to deal with neighbourhood problems and more challenging environments than those without the condition.

## Introduction

An optimal neighbourhood environment is considered to facilitate activity and participation and to contribute to quality of life in old age [1–3]. Previous research has shown that several attributes of the neighbourhood environment are related to the use of neighbourhood resources [4–13]. The influence of the neighbourhood environment on the use of neighbourhood resources may be stronger in older adults with disabilities compared to those without disabilities [1, 14]. Osteoarthritis (OA) of the lower limbs (knees and/or hips) is associated with significant pain and disability in older persons [15, 16]. This study aims to examine the associations between the use of neighbourhood resources and perceived and objective neighbourhood characteristics in older people with and without lower limb osteoarthritis (LLOA) and assesses whether these relationships are stronger in those with the condition.

Theories from environmental gerontology and the World Health Organization’s International Classification of Functioning, Disability and Health suggest that environmental factors can facilitate or impede older adults functioning in terms of activities or participation [17–19]. According to the ecological model of ageing, there is an interaction between individual competence and environmental pressure [17, 18]. Derived from the ecological model of ageing, the environmental docility hypothesis suggests that the less competent the individual, the greater the impact of environmental factors on that individual [17, 18]. Older adults with LLOA may have lower competence than older adults without the condition and may be more vulnerable to environmental demands [20]. Based on the environmental docility hypothesis, perceived and objective characteristics of the neighbourhood environment have a greater impact on older adults with LLOA compared to those without LLOA.

Several perceived and objective characteristics of the neighbourhood have been identified as facilitators and/or barriers for the use of neighbourhood resources. Previous research showed that higher self-perceived neighbourhood cohesion, that is the extent of one’s emotional bond to the neighbourhood [21, 22], was associated with more use of walking areas by older people [4, 10]. Furthermore, people use their neighbourhood environment more when they live in accessible, safe and attractive neighbourhoods, whereas people who perceive more neighbourhood problems, such as crime, litter and traffic, are less likely to use neighbourhood resources [5–9, 11–13]. Moreover, previous research showed that residents of objectively more deprived neighbourhoods make less use of local facilities, such as parks and greenspaces, than those who live in more affluent neighbourhoods [23].

In a previous study using data from the European Project on OSteoArthritis (EPOSA) study, the association between LLOA and the use of the neighbourhood environment was examined [24]. It was found that lower limb OA was associated with less use of parks and walking areas and more use of places to sit and rest. These findings suggest that people with LLOA adjust the use of their neighbourhood environment, and this provides some evidence for the environmental docility hypothesis.

To support activity and promote participation of older adults with LLOA, more knowledge is needed on the relationships between the use of neighbourhood resources and perceived and objective neighbourhood characteristics in this population. This population-based study examined the associations of perceptions of neighbourhood cohesion and neighbourhood problems and objectively measured neighbourhood deprivation with the use of neighbourhood resources by older adults with and without LLOA. It is hypothesised that living in a more deprived neighbourhood and the perception of more neighbourhood problems are associated with less use of neighbourhood resources by older adults. In addition, it is hypothesised that lower levels of perceived neighbourhood cohesion is associated with less use of neighbourhood resources by older persons. It is expected that these associations are stronger in older adults with LLOA than in those without the condition.

## Material and methods

### Design and study sample

The study sample comprised men and women who participated in the United Kingdom (UK) component of the European Project on OSteoArthritis (EPOSA) and who originally participated in the Hertfordshire Cohort Study (HCS). The HCS and the EPOSA study have been described in detail previously [25, 26]. In 1998–2004, men and women born in Hertfordshire (UK), between 1931 and 1939, and still living in the county were recruited to take part in the HCS to evaluate interactions between the genome, the intrauterine and early postnatal environment, and adult diet and lifestyle in the aetiology of chronic disorders in later life [25]. In 2010, a total of 592 participants from the HCS were invited by letter to participate in the EPOSA study. The EPOSA study focuses on the personal and societal burden of OA and its determinants in older adults in six European countries [26]. In total, 444 (75.0 %) persons from HCS agreed to participate in the EPOSA baseline study. Data on perceived neighbourhood cohesion and perceived neighbourhood problems were collected in 2008 using a postal survey. Hertfordshire is a stable county in terms of neighbourhood deprivation [27–29]. In this study, it is assumed that perceptions of neighbourhood cohesion and neighbourhood problems remained stable between 2008 and 2010. The participants who moved between the postal survey (2008) and the EPOSA baseline study (2010) (*n* = 26) were excluded from the analyses. Moreover, those who had missing data on the presence of LLOA (*n* = 17) were omitted. In total, 401 participants were included in the current study. All included participants had data available on the use of neighbourhood resources. For all included participants, data on objectively measured neighbourhood deprivation in 2010 were available. Data from the postal survey on perceived neighbourhood cohesion and perceived neighbourhood problems were available for 303 and 299 participants, respectively. There were no significant differences in characteristics and neighbourhood measures between included and excluded participants (data not shown). In addition, there were no significant differences in characteristics and neighbourhood deprivation between participants who completed the postal survey and those who did not (data not shown). The study was approved by the Hertfordshire Research Ethics Committee.

### Use of neighbourhood resources

In the EPOSA baseline study (2010), use of neighbourhood resources was assessed using a modified version of the Home and Community Environment (HACE) instrument [30]. The HACE is a standardized, self-report instrument designed to assess factors in a person’s environment that may influence levels of participation. The modified version included items pertaining to community mobility and transportation which have been shown to be important features of the neighbourhood environment for older adults with functional limitations [30, 31]. First, the availability of three resources was assessed by asking the participants: ‘Could you please indicate if any of the following facilities can be found in your neighbourhood?’ (1) parks and walking areas that are easy to get to and easy to use; (2) places to sit and rest at bus stops, in parks, or in other places where people walk; (3) public transportation close to home; and (4) public facilities. Response categories were ‘a lot’, ‘some’ and ‘not at all’. When participants answered ‘a lot’ or ‘some’, they were asked whether they made use of the resources (0 = no, 1 = yes).

### Perceived neighbourhood cohesion

In the postal survey (2008), perceived neighbourhood cohesion was assessed using eight items from the 18-item Neighbourhood Cohesion Scale, that was developed to measure sense of community, attraction to neighbourhood and social interaction within it [21, 22, 32, 33]. Examples of items that were included are the following: ‘I feel like I belong to this neighbourhood’ (sense of community), ‘I plan to remain a resident of this neighbourhood for a number of years’ (attraction-to-neighbourhood) and ‘I regularly stop and talk with people in my neighbourhood’ (social interaction within neighbourhood). Participants were asked to indicate how strongly they agreed or disagreed with each statement. Response options ranged from strongly disagree to strongly agree on a 5-point Likert scale. The overall index score ranged from 5 to 40, with higher scores indicating a higher sense of neighbourhood cohesion.

### Perceived neighbourhood problems

In the postal survey (2008), perceived neighbourhood problems were assessed by asking participants to consider a list of eight problems that people often have with the area where they live and indicate whether each one was not a problem (score 1), a small problem (score 2) or a big problem (score 3) for them [33, 34]. The problems were (1) vandalism, (2) litter/rubbish, (3) smells/fumes, (4) assaults/muggings, (5) burglaries, (6) disturbance by children/youngsters, (7) traffic and (8) noise. The overall index score ranged from 8 to 24, with higher scores indicating more problems.

### Objective neighbourhood deprivation

The postal codes of the participants were linked to the 2001 census Lower Super Output Areas (LSOAs) using the GeoConvert online geography matching tool. On average, these LSOAs contain approximately 650 households and 1500 residents [29, 35]. The LSOAs were linked to scores on the Index of Multiple Deprivation 2010 (IMD-2010) [27, 29]. The IMD-2010 provides a measure of area-level multiple deprivation by combining information on seven domains of deprivation, including (1) income, (2) employment, (3) health/disability, (4) education/skills/training, (5) barriers to housing/services, (6) living environment and (7) crime. The lowest and highest IMD-2010 in England were 0.53 and 87.80, respectively. The higher the IMD-2010 score, the more deprived the area of residence.

### Potential effect modifiers

A potential effect modifier was clinical LLOA. In the EPOSA baseline study (2010), the American College of Rheumatology (ACR) classification criteria were used to diagnose OA in the knee and hip [36]. The lower limb OA was defined as present when the participants had clinical OA in the knee and/or hip. An extensive description of the diagnosis of OA in the knee and hip is described elsewhere [26].

### Potential confounders

Potential confounders included age, sex (0 = men, 1 = women), partner status (0 = having no partner, 1 = having a partner), educational level (0 = lower educated than secondary education, 1 = secondary education or a higher level), anxiety, depression, comorbidity and physical activity, which were previously found to be associated with the use of neighbourhood resources and the three neighbourhood measures [37]. Data on these potential confounders were collected in the EPOSA baseline study in 2010.

Anxiety and depressive symptoms were examined by the Hospital Anxiety Depression Scales (HADS) [38]. The HADS is a self-report questionnaire comprising 14 four-point Likert scaled items, 7 for anxiety (HADS-A) and 7 for depression (HADS-D). Both scales ranged from 0 to 21, and a cut-off level of 8 or more was used for presence of anxiety and depression.

Comorbidity was measured through self-reported presence of the following chronic diseases or symptoms that lasted for at least three months or diseases for which the participant had been treated or monitored by a physician: chronic non-specific lung disease, cardiovascular diseases, peripheral artery diseases, stroke, diabetes, cancer and osteoporosis. The number of chronic diseases other than LLOA was categorized into 0, 1, 2 or more chronic diseases.

Physical activity was measured using the Longitudinal Aging Study Amsterdam Physical Activity Questionnaire (LAPAQ) [39]. The LAPAQ estimates the frequency and duration of participation in activities (walking, bicycling, gardening, light and heavy household tasks and sports activities) in the previous 2 weeks, resulting in a total physical activity time in minutes per week.

### Statistical analyses

Characteristics of participants with and without LLOA are presented using descriptive statistics. Differences in means were tested using independent sample *T* tests for normally distributed variables. Differences in medians were tested using the Mann-Whitney U tests for skewed continuous variables, and differences in frequencies were tested using the Pearson Chi-square tests for frequencies.

Logistic regression analyses were used to examine the associations of the three neighbourhood environment measures with the use of neighbourhood resources. First, LLOA was assessed for potential effect modification by examining interaction effects between LLOA and each of the neighbourhood environment measures in fully adjusted models. The interaction effects were considered significant at a *p* value below 0.10 [40]. If a statistically significant interaction term was observed, analyses were stratified for LLOA and group-specific associations between the use of neighbourhood resources and the neighbourhood environment were presented. If the interaction effect was not statistically significant, a pooled analysis (also adjusted for LLOA) was performed. Second, all associations between the use of neighbourhood resources and the neighbourhood environment measures were examined in models constructed step by step. Model 1 examined the association of each neighbourhood environment measure with the use of resources adjusted for sex and age. Model 2 assessed these associations, additionally adjusted for all other confounders. Because of multiple testing, the *p* value was set to 0.01 in all models. Statistical analyses were performed in the IBM SPSS Statistics (version 20.0).

## Results

The characteristics of the participants with and without LLOA are presented in Table 1. The mean age of all 401 participants was 75.2 (SD = 2.6) years with an age-range of 71–80 years. Of all participants, 202 (50.4 %) were female and 74 (18.5 %) persons had LLOA. Of the participants with LLOA, 12.2 % had clinical hip OA, 75.6 % had clinical knee OA and 12.2 % had both knee and hip OA. The proportions of women and depressed as well anxious persons were higher in the LLOA group. Furthermore, participants with LLOA had a lower educational level than those without LLOA.

**Table 1:** Characteristics of the study sample stratified by presence of lower limb osteoarthritis

	All participants (*n* = 401)		Participants with LLOA (*n* = 74)		Participants without LLOA (*n* = 327)		*p* value^a^
	*n*		*n*		*n*		
Characteristics							
Age in years (Mean (SD))	401	75.2 (2.6)	74	75.1 (2.8)	327	75.2 (2.5)	0.63
Sex (female) (*n* (%))	401	202 (50.4)	74	46 (62.2)	327	156 (47.7)	0.03
Partner status (yes) (*n* (%))	401	279 (69.6)	74	51 (68.9)	327	228 (69.7)	0.89
Education (≥secondary education) (*n* (%))	401	322 (80.3)	74	53 (71.6)	327	269 (83.5)	0.04
Number of chronic diseases (*n* (%))	401		74		327		0.36
0		171 (42.6)		32 (43.2)		139 (42.5)	
1		148 (36.9)		23 (31.1)		125 (38.2)	
≥2		82 (20.5)		19 (25.7)		63 (19.3)	
Anxiety (HADS-A ≥8) (*n* (%))	357	58 (16.2)	64	16 (25.0)	293	42 (14.3)	0.04
Depression (HADS-D ≥8) (*n* (%))	362	27 (7.5)	67	13 (19.4)	295	14 (4.7)	<0.001
Physical activity (min/day) (Median (IQR))	399	192.9 (124.3–282.9)	73	171.4 (86.4–225.5)	326	197.1 (129.1–287.1)	0.06

*HADS-A* Hospital Anxiety Depression Scales-Anxiety, *HADS-D* Hospital Anxiety Depression Scales-Depression, *IQR* Interquartile range, *n* number, *LLOA* lower limb osteoarthritis

^a^ *p* value of observed differences between groups with and without LLOA

### Use of neighbourhood resources

Most participants reported that they have a lot or some availability of parks and walking areas, places to sit and rest, public transport and public facilities in their neighbourhood. Of those participants, the majority reported that they made use of parks and walking areas, public transport and public facilities. Most participants reported that they did not make use of places to sit and rest. No differences were found between older people with and without LLOA in the availability of neighbourhood resources. Participants with LLOA reported making more use of places to sit and rest than their counterparts without LLOA (Table 2).

**Table 2:** Availability and use of four types of neighbourhood resources in the study sample stratified by the presence of lower limb osteoarthritis

	All participants (*n* = 401)		Participants with LLOA (*n* = 74)		Participants without LLOA (*n* = 327)		*p* value^a^
	*n*		*n*		*n*		
Availability of neighbourhood resources							
Availability of parks and walking areas (a lot/some) (*n* (%))	401	379 (94.5)	74	69 (93.2)	327	310 (94.8)	0.60
Availability of places to sit and rest (a lot/some) (*n* (%))	399	361 (90.5)	74	67 (90.5)	325	294 (90.5)	0.98
Availability of public transport (a lot/some) (*n* (%))	400	381 (95.3)	74	70 (94.6)	326	311 (95.4)	0.77
Availability of public facilities (a lot/some) (*n* (%))	401	373 (93.0)	74	70 (94.6)	327	303 (92.7)	0.56
Use of neighbourhood resources							
Parks and walking areas (yes) (*n* (%))	379	242 (63.9)	69	43 (62.3)	310	199 (64.2)	0.77
Places to sit and rest (yes) (*n* (%))	361	154 (42.7)	67	41 (61.2)	294	113 (38.4)	<0.01
Public transport (yes) (*n* (%))	381	196 (51.4)	70	41 (58.6)	311	155 (49.8)	0.19
Public facilities (yes) (*n* (%))	373	338 (90.6)	70	62 (88.6)	303	276 (91.1)	0.52

*n* number, *LLOA* lower limb osteoarthritis

^a^ *p* value of observed differences between groups with and without LLOA

### Neighbourhood environment

The characteristics of the neighbourhood environment are presented in Table 3. In the full sample, the average perceived neighbourhood cohesion score was 32.2 (SD = 4.5) and the average perceived neighbourhood problem index was 11.0 (SD = 2.6). In the full sample, the IMD-2010 score ranged from 1.45 to 34.72, with a median (interquartile range (IQR)) score of 9.09 (5.08–13.29).

**Table 3:** Characteristics of the neighbourhood environment in the study sample stratified by the presence of lower limb osteoarthritis

	All participants (*n* = 401)		Participants with LLOA (*n* = 74)		Participants without LLOA (*n* = 327)		*p* value^a^
	*n*		*n*		*n*		
Neighbourhood environment							
Perceived neighbourhood cohesion (5–40) (Mean (SD))	303	32.2 (4.5)	53	32.3 (5.5)	250	32.2 (4.3)	0.82
Perceived neighbourhood problems (8–24) (Mean (SD))	299	11.0 (2.6)	51	12.0 (2.6)	248	10.9 (2.5)	<0.01
Objective neighbourhood deprivation (Median (IQR))	401	9.09 (5.08–13.29)	74	9.42 (6.26–18.42)	327	9.09 (4.92–13.16)	0.95

*n* number, *IQR* interquartile range, LLOA lower limb osteoarthritis

^a^ *p* value of observed differences between groups with and without LLOA

Participants with LLOA perceived significantly more neighbourhood problems compared to their counterparts without LLOA (LLOA: Mean = 12.0, SD = 2.6 versus non-LLOA: Mean = 10.9, SD = 2.5; *p* < 0.01). Perceived neighbourhood cohesion and objective neighbourhood deprivation did not differ between participants with and without LLOA (Table 3).

### Use of neighbourhood resources and perceived neighbourhood cohesion

After adjustment for all confounders, a trend for a positive association between perceived neighbourhood cohesion and use of places to sit and rest were observed in the full sample (OR = 1.07, 99 % CI = 0.98–1.16) (Table 4; model 2). The associations between perceived neighbourhood cohesion and the use of neighbourhood resources did not differ between participants with and without LLOA.

**Table 4:** Associations between characteristics of the neighbourhood environment and the use of neighbourhood resources by older adults

	Use of parks and walking areas	Use of places to sit and rest	Use of public transportation	Use of public facilities
	OR (99 % CI)	OR (99 % CI)	OR (99 % CI)	OR (99 % CI)
Perceived neighbourhood cohesion				
Model 1	1.04 (0.97–1.12)	1.03 (0.96–1.11)	1.00 (0.93–1.07)	0.98 (0.88–1.10)
Model 2	1.04 (0.95–1.13)	1.07 (0.98–1.16)^*^	0.98 (0.91–1.07)	0.96 (0.84–1.09)
Perceived neighbourhood problems				
Model 1	0.98 (0.86–1.11)	1.02 (0.90–1.16)	1.12 (0.98–1.27)^**^	1.02 (0.83–1.24)
Model 2	0.98 (0.84–1.13)	0.98 (0.85–1.13)	1.10 (0.96–1.26)^*, a^	1.01 (0.81–1.25)
Objective neighbourhood deprivation				
Model 1	1.00 (0.95–1.04)	1.03 (0.98–1.07)	1.02 (0.98–1.06)	0.96 (0.90–1.02)^*^
Model 2	1.00 (0.96–1.06)	1.03 (0.98–1.08)	1.02 (0.98–1.07)	0.96 (0.89–1.03)

Model 1: adjusted for age and sex (reference category: men)

Model 2: additionally adjusted for partner status (reference category: no partner), educational level (reference category: lower educated than secondary education), socio-economic status (reference category: routine occupations), anxiety (reference category: not anxious), depression (reference category: not depressed), number of chronic diseases (reference category: no chronic diseases other than lower limb osteoarthritis (LLOA)), physical activity and LLOA (reference category: no LLOA)

*OR* odds ratio, *CI* confidence interval

^***^ *p* < 0.01, ^**^0.01 ≥ *p* < 0.05, ^*^0.05 ≥ *p* < 0.10

^a^There was a significant LLOA by perceived neighbourhood problems interaction effect on the use of public transport. Therefore, the association in this model was not additionally adjusted for LLOA

### Use of neighbourhood resources and perceived neighbourhood problems

**Fig. 1:**
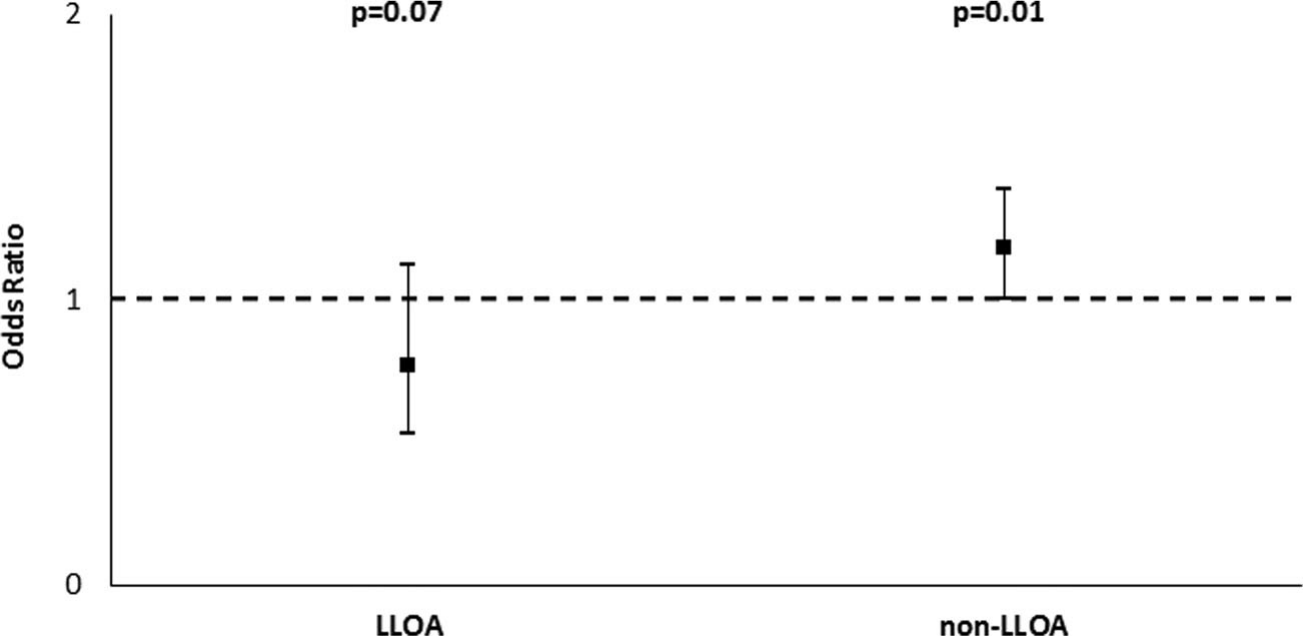
**Association between the use of public transport and perceived neighbourhood problems in older adults with and without lower limb osteoarthritis**. *LLOA* lower limb osteoarthritis. The odds ratio of perceived neighbourhood problems is presented. *Error bars* represent 99 % confidence intervals. The associations are adjusted for age, sex (reference category: men), partner status (reference category: no partner), educational level (reference category: not better educated than secondary education), anxiety (reference category: not anxious), depression (reference category: not depressed), number of chronic diseases (reference category: no chronic diseases other than lower limb osteoarthritis) and physical activity

After adjustment for all confounders, no statistically significant associations between perceived neighbourhood problems and use of neighbourhood resources were observed in the full sample (Table 4; model 2). A significant LLOA by perceived neighbourhood problems interaction effect on the use of public transport was observed (*p* = 0.03). A trend for a negative association between perceived neighbourhood problems and use of public transport was observed in participants with LLOA (OR = 0.77, 99 % CI = 0.53–1.12), whereas a trend for a positive association between perceived neighbourhood problems and use of public transport was found in those without LLOA (OR = 1.18, 99 % CI = 1.00–1.39) (Fig. 1).

### Use of neighbourhood resources and objective neighbourhood deprivation

After adjustment for all confounders, no statistically significant associations between objective neighbourhood deprivation and use of neighbourhood resources were observed in the full sample (Table 4; model 2). The associations between neighbourhood deprivation and the use of resources did not differ between participants with and without LLOA.

## Discussion

This study examined the associations of perceptions of neighbourhood cohesion and neighbourhood problems and objectively measured neighbourhood deprivation with the use of neighbourhood resources in older adults with and without LLOA living in Hertfordshire, UK, and assessed whether these relationships were stronger in older persons with LLOA than in those without the condition. It was found that, regardless of LLOA, perceived neighbourhood cohesion and objective neighbourhood deprivation were not significantly associated with use of resources by older adults. Furthermore, the results showed that perception of more neighbourhood problems was marginally significantly associated with more use of public transport in older adults without LLOA, whereas the perception of more neighbourhood problems was marginally significantly associated with less use of public transport in older adults with LLOA.

Based on the environmental docility hypothesis [17, 18], it was expected that lower levels of perceived neighbourhood cohesion, more perceived neighbourhood problems and higher levels of objectively measured neighbourhood deprivation would be associated with less use of resources in older adults and that these associations would be stronger in older persons with LLOA than in those without the condition. In contrast with the environmental docility hypothesis [17, 18], the results of this study showed that, regardless of LLOA, perceived neighbourhood cohesion and objective neighbourhood deprivation were not significantly associated with use of resources by older adults. In line with the environmental docility hypothesis [17, 18], it was found that older adults with LLOA perceive more neighbourhood problems than those without LLOA. This suggests that older adults with LLOA might be more vulnerable to environmental demands than those without the condition, due to the experience of more pain and disability. The findings also showed that the perception of more neighbourhood problems was associated with more use of public transport in older adults without LLOA, whereas older adults with LLOA were less likely to make use of public transport when they perceive more neighbourhood problems. In line with the environmental docility hypothesis [17, 18], the perception of more neighbourhood problems seems to hinder older adults with LLOA to make use of public transport facilities. The perception of more neighbourhood problems seems not to be a barrier for older adults without LLOA to make use of these neighbourhood resources. These findings suggest that older adults with LLOA may be less able to deal with perceived neighbourhood problems and more challenging environments than those without LLOA. Older adults with LLOA may reduce their use of public transport, because they do not want to travel through their neighbourhood to public transport facilities and be exposed to their perceived neighbourhood problems. However, the results do not show any association between perceived neighbourhood problems and use of other neighbourhood resources in older adults with LLOA.

Perception of more neighbourhood problems seems to hinder older adults with LLOA to make use of public transport and this may have an important negative impact on their daily functioning. In a study by Martin et al., community-dwelling older adults with OA identified public transport as an important community resource that they use to manage their OA as it facilitates easier access to public services and health care resources [41]. In addition, previous studies suggest that public transport is an important resource for older persons to maintain social relationships, personal independence and participation in activities [42, 43].

Older adults with LLOA reported making more use of places to sit and rest in their neighbourhood than their counterparts without LLOA. Individuals with LLOA might be more dependent on these amenities, because they experience more pain and disability than those without the condition and, as a consequence, they may need to rest more often during their outdoor activities. Regardless of LLOA, a trend for a positive association between perceived neighbourhood cohesion and use of places to sit and rest were observed. Previous research showed that more availability of places to sit and rest results in more use of these resources [24]. It could be that the availability of places to sit and rest in a neighbourhood increases the use of these places by residents, which may facilitate attractiveness of a neighbourhood, social interaction within a neighbourhood and a higher sense of community among residents in a neighbourhood. Another possible explanation for this finding could be that older adults with a higher sense of neighbourhood cohesion are more likely to make use of places to sit and rest, because they can spend time outside and meet other residents of their neighbourhood.

The current study extends previous research by examining the association between use of resources and characteristics of the immediate neighbourhood environment measured through self-reports and objective assessments in older adults with and without LLOA. This study has several strengths, including extensive phenotyping of study participants according to strict study protocols and by a highly trained research team.

Some limitations have to be acknowledged as well. Neighbourhood cohesion and problems were measured in 2008, whereas the assessment of LLOA, use of resources and the covariates were assessed in 2010. It has been assumed that perceptions of neighbourhood cohesion and problems remained stable between 2008 and 2010. Although Hertfordshire is a stable county in terms of deprivation [27–29] and people who moved within this period were excluded from the analyses, the 2-year lag and the small sample size might have made it harder to gauge the true size of the associations between the neighbourhood environment and use of resources by older people with and without LLOA. Furthermore, the cross-sectional design makes it impossible to be certain about the direction of effect of sense of neighbourhood cohesion and perceptions of neighbourhood problems on use of neighbourhood resources. Another limitation is related to the geographical distribution of the study sample. The study sample is drawn from a single county which has low levels of deprivation compared to other parts of the UK [27, 29]. Moreover, the participants in this study cannot be considered typical of all men and women of this age in the UK, because they have continued to live in the county of their birth [25]. However, participants of the HCS have been shown to be very similar to those in the national representative Health Survey for England on a range of characteristics [25].

This study is limited to perceived availability and use of some neighbourhood resources. Future research could focus on objectively measured availability and actual use of neighbourhood resources by using objective data on the built environment and by using Global Position System (GPS) devices. Furthermore, future research should not only focus on the neighbourhood resources that were included in the HACE instrument but also need to consider other neighbourhood resources that are important for older adults with and without LLOA. Future research could also consider other relevant perceived and objective neighbourhood characteristics, such as hilly terrain, accessibility of buildings, and poor pavement conditions. In addition, future studies with larger study samples are needed to appropriately investigate the association between use of neighbourhood resources and neighbourhood characteristics. Moreover, longitudinal, prospective studies are needed to investigate the causal relationships of perceptions of neighbourhood cohesion and neighbourhood problems and objectively measured neighbourhood deprivation with the use of neighbourhood resources by older adults with and without LLOA.

In conclusion, the results of the current study provide limited supportive evidence for the environmental docility hypothesis. Regardless of LLOA, perceived neighbourhood cohesion and objective neighbourhood deprivation were not significantly associated with use of resources by older adults. The perception of more neighbourhood problems seems only to hinder older adults with LLOA to make use of public transport facilities, but not of other neighbourhood resources. Older adults with LLOA may be less able to deal with neighbourhood problems and more challenging environments than those without the condition and may be, therefore, more likely to reduce their use of public transport when they perceive more neighbourhood problems.
